# Evaluation of antenatal risk factors for postpartum depression: a secondary cohort analysis of the cluster-randomised GeliS trial

**DOI:** 10.1186/s12916-020-01679-7

**Published:** 2020-07-24

**Authors:** Hamimatunnisa Johar, Julia Hoffmann, Julia Günther, Seryan Atasoy, Lynne Stecher, Monika Spies, Hans Hauner, Karl-Heinz Ladwig

**Affiliations:** 1 Institute of Epidemiology, Helmholtz Zentrum München, German Research Center for Environmental Health, Ingolstädter Landstraße 1, 85764 Neuherberg, Germany; 2 Department of Psychosomatic Medicine and Psychotherapy, Justus-Liebig University of Giessen and Marburg, Giessen, Friedrichstr. 33, 35392 Gießen, Germany; 3 Institute of Nutritional Medicine, Else Kröner-Fresenius-Centre for Nutritional Medicine, Klinikum rechts der Isar, Technical University of Munich, Georg-Brauchle-Ring 62, 80992 Munich, Germany; 4 Department of Psychosomatic Medicine and Psychotherapy, Klinikum rechts der Isar, Technische Universität München, Langerstr. 3, 81675 Munich, Germany

**Keywords:** Postpartum depression, Anxiety, Obesity prevention, Routine care, Gestational weight gain, Lifestyle intervention, EPDS, Well-being, Predictor

## Abstract

**Background:**

Maternal weight variables are important predictors of postpartum depression (PPD). While preliminary evidence points to an association between pre-pregnancy obesity and PPD, the role of excessive gestational weight gain (GWG) on PPD is less studied. In this secondary cohort analysis of the German ‘healthy living in pregnancy’ (GeliS) trial, we aimed to investigate associations between weight-related variables and PPD and to assess the influence of GWG on the risk for PPD.

**Methods:**

We included women with normal weight, overweight, and obesity (BMI 18.5–40.0 kg/m^2^). Symptoms of PPD were assessed 6–8 weeks postpartum using the Edinburgh Postnatal Depression Scale. Pre-pregnancy BMI was self-reported. During the course of pregnancy, weight was measured at gynaecological practices within regular check-ups. GWG was defined as the difference between the last measured weight before delivery and the first measured weight at the time of recruitment (≤ 12^th^ week of gestation). Excessive GWG was classified according to the Institute of Medicine. Multiple logistic regression analyses were used to estimate the odds of PPD in relation to pre-pregnancy BMI, GWG, and excessive GWG adjusting for important confounders.

**Results:**

Of the total 1583 participants, 45.6% (*n* = 722) showed excessive GWG and 7.9% (*n* = 138) experienced PPD. Pre-pregnancy BMI (per 5-unit increase; OR = 1.23, 95% CI 1.08–1.41, *p* = 0.002) and pre-pregnancy overweight or obesity were significantly positively associated with the odds of developing PPD, particularly among women with an antenatal history of anxiety or depressive symptoms (overweight: OR = 1.93, 95% CI = 1.15–3.22, *p* = 0.01; obesity: OR = 2.11, 95% CI = 1.13–3.96, *p* = 0.02). Sociodemographic or lifestyle factors did not additively influence the odds of having PPD. In fully adjusted models, there was no significant evidence that GWG or the occurrence of excessive GWG increased the odds of experiencing PPD (excessive vs. non-excessive: OR = 3.48, 95% CI 0.35–34.94; GWG per 1 kg increase: OR = 1.16, 95% CI 0.94–1.44).

**Conclusion:**

Pre-pregnancy overweight or obesity is associated with PPD independent of concurrent risk factors. History of anxiety or depressive symptoms suggests a stress-induced link between pre-pregnancy weight and PPD.

**Trial registration:**

NCT01958307, ClinicalTrials.gov, retrospectively registered on 9 October 2013.

## Background

Postpartum depression (PPD) is a mental health complication that can occur after childbirth [1–3] with prevalence estimates ranging from 10 to 15% worldwide [4, 5] and 3 to 6% for Germany [6, 7]. PPD is characterised by the mother’s fear of failure, low mood, emotional ambivalence, and inability to experience pleasure, which are often presented with additional symptoms of major depressive disorders [1, 2, 4]. The occurrence of depressive symptoms has been linked to an impaired maternal caregiving behaviour, leading to disturbed mother-to-infant attachment [4, 8–10]. Hence, PPD not only affects maternal health, but can also negatively influence the mother-infant relationship as well as the long-term development of the child [4, 10].

The aetiology of PPD is still not completely understood [11, 12]. In the last years, considerable efforts have been made to identify predictors and early modifiable risk factors of PPD. Research in this field could increase the success of PPD management and ultimately advance our proceedings in the early prevention of PPD and associated maternal and infant complications.

In this context, a possible association between maternal weight and onset of PPD continues to receive increasing awareness, although the evidence remains limited and inconclusive. While some studies have found an association between pre-pregnancy overweight or obesity and PPD [13–16], others failed to confirm these findings [17, 18]. In addition to maternal pre-pregnancy weight status, the role of excessive gestational weight gain (GWG) as a risk factor of adverse maternal outcomes has recently been highlighted [19]. However, the influence of GWG and excessive GWG on the incidence of PPD has rarely been examined. The current state of research indicates no consistent association between GWG or excessive GWG and PPD [15–17, 20–22]. Most studies evaluating the influence of body weight or GWG on PPD were limited by small sample size and the inability to control for a range of confounding factors, in particularly the history of depressive symptoms during pregnancy. Therefore, further investigations are needed to disentangle the influence of weight-related variables on the development of PPD. This is fundamental to improve the screening for early risk factors of PPD alongside primary care and ultimately to advance in the prevention of PPD itself and associated adverse outcomes.

Using data from the German cluster-randomised ‘Gesund leben in der Schwangerschaft’/‘healthy living in pregnancy’ (GeliS) study, we herein aim to outline current inconsistencies. The GeliS trial was initially designed to reduce the proportion of women with excessive GWG and to prevent adverse health outcomes such as PPD by providing pregnant women with a comprehensive lifestyle intervention alongside the German routine care [23]. The GeliS intervention was neither successful in reducing the proportion of women with excessive GWG [24], nor influenced the maternal postpartum weight development substantially [25]. However, the intervention resulted in small to moderate improvements in maternal dietary [26] and physical activity behaviour [27]. Further, the GeliS study included a large sample of pregnant women with extensive data on maternal health and used a validated tool for assessing PPD. Thus, it is valuable to investigate determinants of PPD from different angles.

The present analysis aimed to examine the associations between pre-pregnancy BMI or GWG and PPD in the pooled GeliS cohort. Furthermore, we examined how the history of anxiety or depressive symptoms during pregnancy may modify a potential association taking various sociodemographic, lifestyle, and clinical factors into consideration.

## Methods

### Study setting and population

The GeliS study is a prospective, multicentre, cluster-randomised, controlled, open intervention trial that primarily aimed to reduce the proportion of women with excessive GWG as defined by the US Institute of Medicine (IOM) [28]. Secondary aims were to reduce the risk for adverse perinatal and postpartum complications, such as PPD, and to improve behavioural outcomes, such as physical activity, dietary, and breastfeeding behaviour [23]. Details about the design, setting, population, and randomisation process have been described elsewhere [23].

In brief, women with (1) a pre-pregnancy BMI between ≥ 18.5 kg/m^2^ and ≤ 40.0 kg/m^2^, (2) a singleton pregnancy, (3) age between 18 and 43 years, (4) sufficient German language skills, and (5) stage of pregnancy before the end of the 12^th^ week of gestation were recruited between 2013 and 2015. The recruitment was conducted in gynaecological and midwifery practices in five administrative regions of Bavaria (Germany) depicting the ‘real-life’ setting of antenatal routine care. All participants gave their written informed consent for participation.

Participants in the control group obtained routine antenatal care and additionally general information on a healthy antenatal lifestyle by means of a flyer. Participants in the intervention group received a comprehensive lifestyle intervention programme. This programme consisted of three antenatal and one postpartum face-to-face counselling sessions on a healthy pre- and postnatal lifestyle according to current recommendations for the antenatal and postpartum period [29–31]. The counselling sessions were given by previously trained midwives, medical personnel, or gynaecologists alongside routine care visits. Details on the counselling content have already been reported [23].

The study was performed in accordance with the current local regulatory requirements and the Declaration of Helsinki. The Ethics Commission of the Technical University of Munich approved the study protocol. The trial is registered at the ClinicalTrials.gov Protocol Registration System (NCT01958307).

### Data collection and outcomes

All baseline characteristics including sociodemographic information were collected at the time of recruitment (before the end of the 12^th^ week of gestation) using a screening questionnaire. Pre-pregnancy BMI was calculated based on the self-reported pre-conception weight. Having a BMI between 18.5 and 24.9 kg/m^2^ was defined as being normal weight, between 25.0 and 29.9 kg/m^2^ as being overweight, and between 30.0 and 40.0 kg/m^2^ as having obesity. Based on details on the educational level collected via the screening questionnaire, participants were grouped into having a ‘lower educational level’ if they at least completed high school and into the ‘higher educational level’ category if they held a university degree.

During the course of pregnancy, maternal weight data were collected by means of routinely used maternity records. GWG was defined as the difference between the last measured weight before delivery and the first measured weight at the time of recruitment. Excessive GWG was defined according to the thresholds provided by the IOM [28] considering the woman’s pre-pregnancy BMI category. The optimal GWG ranges for women with normal weight were 11.5–16.0 kg, for women with overweight 7.0–11.5 kg, and for women with obesity 5.0–9.0 kg. Gaining weight above these thresholds was defined as excessive GWG [28]. Between the 24 and 28 weeks of gestation, a 2-h oral glucose tolerance test was performed for the screening and diagnosis of gestational diabetes mellitus. According to national and international recommendations [32, 33], gestational diabetes mellitus was diagnosed if one of the following thresholds was equalled or exceeded: fasting plasma glucose, 92 mg/dL (5.1 mmol/L); 1-h value, 180 mg/dL (10.0 mmol/L); and 2-h value, 153 mg/dL (8.5 mmol/L). Pre-pregnancy or early pregnancy lifestyle factors, such as smoking status, physical activity level, intake of alcohol, and mental health state, were inquired in a set of questionnaires that was answered by participants directly after inclusion (before the end of the 12^th^ week of gestation). This set of questionnaires contained a slightly modified version of the validated food frequency questionnaire developed for the ‘German Health Examination Survey for Adults’ (DEGS) study by the Robert Koch Institute, Berlin, Germany [34], which was used to group women according to ‘any alcohol consumption’ and ‘no alcohol consumption’. Moreover, it comprised the validated ‘Pregnancy Physical Activity Questionnaire’ (PPAQ) [35] that was slightly adapted to German habits. Thereby, participants had to estimate the mean time spent engaging in 32 activities in the past month. As described in the evaluation instructions of this questionnaire [35], calculated average weekly energy expenditure in MET-h/week was summed up into the category ‘total physical activity of light intensity and above’. The median MET-h/week value of this variable was used to group participants into having a ‘low level of physical activity’ or a ‘high level of physical activity’. Furthermore, the set of questionnaires comprised questions of anxiety and depressive symptoms by using validated ‘Patient Health Questionnaire for Depression and Anxiety’ (PHQ-4). It comprised four items with a 4-point scale to screen for depression and anxiety. The composite PHQ-4 total score ranges from 0 to 12, and scale scores of ≥ 3 were suggested as cut-off points of probable cases of depression or anxiety [36].

Between 6 and 8 weeks postpartum, symptoms of PPD were assessed using the validated German version of the ‘Edinburgh Postnatal Depression Scale’ (EPDS) [37]. Showing symptoms of PPD was defined as having an EPDS score ≥ 13 and is in the following described as ‘having PPD’.

### Statistical analyses

A power calculation was performed based on the primary study outcome excessive GWG and was described elsewhere [23]. We found no between-group differences in the history of anxiety and depressive symptoms in early pregnancy or in the history of PPD (Additional file 1). Therefore, we made the post hoc decision to pool data from both the intervention and control groups and considered the group assignment as a covariate.

We performed complete-case analyses as defined a priori [24] and included participants with GWG data, EPDS data, and covariate data available. We excluded those participants who had a preterm delivery (< 37^th^ delivery). All statistical analyses were performed in SAS version 9.4 (SAS Institute Inc., Cary, NC), and *p* values below 0.05 were considered as statistically significant.

#### Participant characteristics

Participant characteristics including sociodemographic, lifestyle, clinical, and psychological characteristics are presented for the total cohort and then stratified according to PPD status. Categorical variables are summarised as proportions and compared between the PPD and non-PPD groups using *χ*^2^ tests. Continuous variables are summarised as mean ± standard deviation (SD) and compared between the PPD and non-PPD groups using Kruskal-Wallis tests. Similar analyses were performed to compare characteristics between the excessive/non-excessive GWG groups and pre-pregnancy BMI categories.

#### Association between pre-pregnancy BMI and PPD

Multivariable logistic regression models were fit to assess the association between pre-pregnancy BMI and PPD. Due to clusters in the dataset (the randomised regions in the trial), the models were fit with generalised estimating equations. Models were fitted with different levels of adjustment. Model 1 was adjusted for age and group allocation. Model 2 was further adjusted for marital status, educational level, and parity. Model 3 was additionally adjusted for smoking status, alcohol intake, and the level of physical activity. Model 4 was further adjusted for history of anxiety or depressive symptoms during early pregnancy. Models 1–4 were fit with BMI both as a continuous and categorical variable. Model results are presented as odds ratios (ORs) with 95% confidence intervals (95% CIs). For continuous BMI, the odds ratio and 95% CIs are presented for each 5-unit increment in pre-pregnancy BMI. To determine the best fitting model, we computed the ‘quasi-likelihood under the independence’ model criterion (QIC).

#### Association between GWG or excessive GWG and PPD

To assess the association between GWG or excessive GWG and PPD, analogous Models 1–4 were fitted. All models were additionally adjusted for pre-pregnancy BMI and the interaction term between pre-pregnancy BMI and GWG or excessive GWG, as previously recommended [38]. Models were fitted with GWG as a continuous variable or excessive GWG as a categorical variable. In the continuous case, odds ratios are presented for each 1 kg increase in GWG. To determine the best fitting model, we computed the QIC statistic.

Further, we explored a non-linear association between continuous GWG and PPD using restricted cubic splines. Additional regression analyses were conducted to investigate the specific association between excessive or inadequate GWG (adequate GWG as the reference category) and the odds of having PPD.

#### Sensitivity analyses

Further logistic regression models were used to assess if antenatal anxiety or depressive symptoms modify the effect of BMI or GWG on the odds for PPD. The interaction terms of history of anxiety or depressive symptoms by pre-pregnancy BMI or GWG on the risk for PPD were added to model 4.

## Results

### Descriptive analysis

**Fig. 1:**
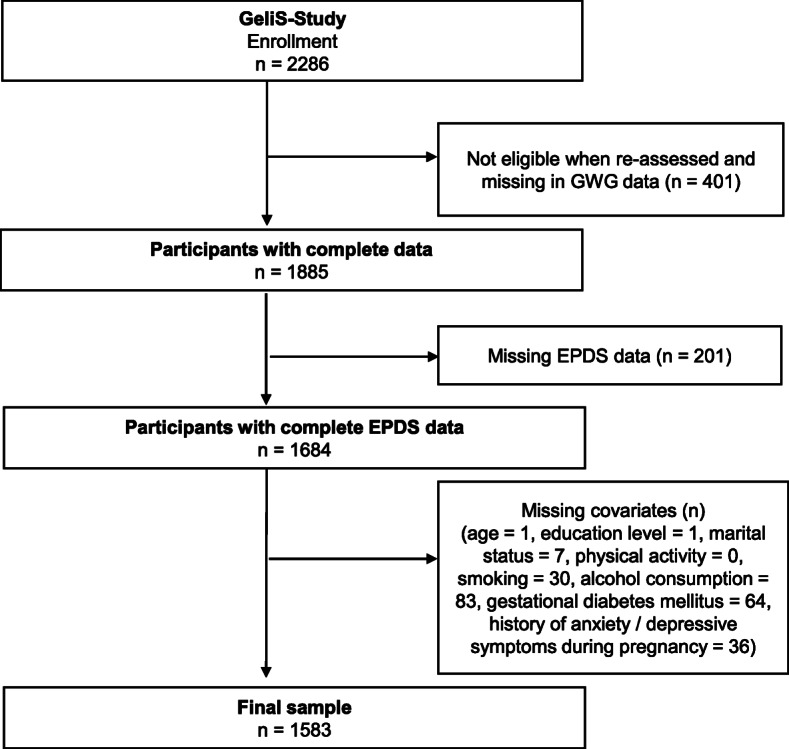
Flowchart of included study participants. EPDS, Edinburgh Postnatal Depression Scale; GDM, gestational diabetes mellitus; GWG, gestational weight gain

Overall, 2286 women were enrolled in the GeliS study. Among them, 1684 were eligible for the present analyses and 1583 of them provided information on all covariates (Fig. 1). Excluded participants differed in terms of mean GWG, parity, history of gestational diabetes, marriage status, smoking, and physical activity habits from the study sample as outlined in Additional file 2.

The baseline study population had a mean age of 30.4 ± 4.4 years (Table 1). In total, 1047 (66.1%) women were in the normal weight category, 352 (22.2%) had a BMI between 25.0 and 29.9 kg/m^2^ and thus overweight, and 184 (11.6%) had a BMI ≥ 30.0 kg/m^2^ and thus obesity (Table 1). In the postpartum period, 7.9% (*n* = 138) participants had PPD among whom 16.7% (*n* = 23) had obesity and 53.6% (*n* = 74) had excessive GWG. The prevalence of overweight and obesity was higher in the subgroup of women with PPD compared to women without PPD (Table 1). Moreover, the rate of excessive GWG tended to be higher in women with PPD (Table 1). Table 1 summarises the sociodemographic, lifestyle, metabolic, and psychological characteristics of the participants according to PPD status. Participants who experienced PPD were more likely to have a lower educational level, be unmarried, smoke during early pregnancy, and suffer from antenatal anxiety and depressive symptoms. Moreover, the proportion of women with a university degree was lower in the subgroup of women with PPD. There were no significant differences in age, parity, alcohol consumption, living conditions, physical activity level, and gestational diabetes mellitus status between women with and without PPD.

**Table 1:** Characteristics (*n* (%)) of study participants according to PPD status

	**Total** ***n*** **= 1583**		**PPD** ***n*** **= 138 (7.9%)**	**No PPD** ***n*** **= 1445 (92.2%)**	*p*value*
**Maternal characteristics**					
Pre-pregnancy BMI, mean ± SD	24.3 ± 4.4		25.2 ± 4.8	24.2 ± 4.4	**0.01**
Pre-pregnancy BMI category					**0.01**
BMI 18.5–24.9 kg/m^2^	1047 (66.1)		74 (53.6)	973 (67.3)	
BMI 25.0–29.9 kg/m^2^	352 (22.2)		41 (29.7)	311 (21.5)	
BMI 30.0–40.0 kg/m^2^	184 (11.6)		23 (16.7)	163 (11.1)	
Excessive GWG	722 (45.6)		74 (53.6)	648 (44.9)	< 0.05
Parity					0.10
0	930 (58.8)		93 (67.4)	837 (57.9)	
1	536 (33.9)		37 (26.8)	499 (34.5)	
≥ 2	117 (7.4)		8 (5.8)	109 (7.5)	
**Demographic factors**					
Age, mean ± SD	30.4 ± 4.4		29.8 ± 4.7	30.4 ± 4.4	0.10
Educational level					
High school or others	930 (58.8)		93 (67.4)	837 (57.9)	**0.03**
University	653 (41.3)		45 (32.6)	608 (42.1)	
Married	1057 (66.8)		76 (55.1)	981 (67.9)	**< 0.01**
Living alone	47 (3.0)		7 (5.1)	40 (2.8)	0.13
**Lifestyle and metabolic factors**					
Alcohol consumption	481 (30.4)		47 (34.1)	434 (30.0)	0.33
Smoking	80 (5.1)		13 (9.4)	67 (4.6)	**0.01**
Low level of physical activity°	802 (50.9)		72 (52.2)	730 (50.5)	0.71
Gestational diabetes mellitus	155 (10.2)		18 (13.4)	137 (9.9)	0.19
**Psychological factors**					
Antenatal history of anxiety/depressive symptoms°°	60 (41.7)		94 (68.1)	566 (39.2)	**< .0001**

*Abbreviations*: *BMI* body mass index, *GWG* gestational weight gain (as defined by the IOM)

*p value for differences between PPD vs. non-PPD using the Kruskal-Wallis test for continuous variables and the *χ*^2^ test for categorical variables

°Assessed by the Pregnancy Physical Activity Questionnaire (PPAQ) before the end of the 12^th^ week of gestation

°°Assessed by the Patient Health Questionnaire for Depression and Anxiety (PHQ)-4 before the end of the 12^th^ week of gestation

Women with obesity had a lower GWG in comparison to women with normal weight or overweight (mean ± SD: obesity, 11.0 ± 6.7 kg; overweight, 14.0 ± 5.7 kg; normal weight, 14.7 ± 4.5 kg; *p* < .001). Additional file 3 shows the characteristics of women according to their pre-pregnancy BMI category.

Overall, 45.6% (*n* = 722) of participants showed excessive GWG according to the IOM criteria. The proportion of overweight and obesity was higher in the subgroup of women with excessive GWG compared to non-excessive GWG counterparts. This also applied for having a history of antenatal anxiety and depressive symptoms (Additional file 4).

### Association between pre-pregnancy BMI and PPD

Table 2 shows the multivariable regression models for the association between pre-pregnancy BMI and PPD presented with the odds ratios (ORs) and 95% CIs. Pre-pregnancy BMI (per 5-unit increment) was positively associated with the odds of experiencing PPD (model 1: OR = 1.25, 95% CI = 1.10–1.44, *p* = 0.03), indicating that a 5-kg/m^2^ increase in BMI corresponded to a 25% increase in the odds of having PPD. The association between pre-pregnancy BMI and PPD remained stable after adjusting for concurrent risk factors (full model OR = 1.23, 1.08–1.41, *p* = 0.002). Being married significantly decreased the odds of PPD (model 4: OR = 0.70, 95% CI = 0.54–0.91, *p* = 0.04), whereas a low educational level was positively associated with the odds of PPD (model 4: OR = 1.41, 95% CI = 1.02–1.94, *p* = 0.01). Among all, history of anxiety or depressive symptoms led to the highest odds of experiencing PPD (full model OR = 3.42, 95% CI = 2.42–4.82, *p* < .001). There was no significant evidence for associations between additional sociodemographic and lifestyle factors or GDM status and the odds of experiencing PPD symptoms. The QIC statistics revealed that the final model was the preferred model fit indicated by the smallest QIC values (data not shown).

**Table 2:** Associations between pre-pregnancy BMI (per 5-unit increase) and PPD at 6–8 weeks postpartum (*n* = 1583)

**Covariate**	**Model 1**	**Model 2**	**Model 3**	**Model 4**
**Pre-pregnancy BMI**	1.25 (1.10–1.44)*	1.23 (1.07–1.41)**	1.23 (1.07–1.41)**	1.23 (1.08–1.41)**
Age	0.96 (0.93–1.00)*	0.99 (0.95–1.03)	0.99 (0.96–1.02)	1.00 (0.97–1.04)
Group allocation	1.34 (0.90–2.02)	1.38 (0.93–2.05)	1.39 (0.95–2.05)	1.39 (0.93–2.09)
Married		0.62 (1.06–1.99)*	0.66 (0.50–0.88)*	0.70 (0.54–0.91)*
Lower educational level		1.46 (0.48–0.80)**	1.40 (1.00–1.96)*	1.41 (1.02–1.94)*
Parity				
1		0.75 (0.49–1.14)	0.74 (0.51–1.08)	0.73 (0.51–1.04)
≥ 2		0.80 (0.45–1.41)	0.74 (0.42–1.31)	0.66 (0.37–1.20)
Alcohol intake			1.18 (0.86–1.64)	1.21 (0.86–1.69)
Low level of physical activity°			0.99 (0.73–1.34)	0.97 (0.72–1.30)
Smoking			1.81 (0.95–3.44)	1.59 (0.82–3.10)
Gestational diabetes mellitus			1.17 (0.84–1.65)	1.23 (0.90–1.70)
Antenatal history of anxiety/depressive symptoms°°				3.42 (2.42–4.82)***

Depicted are odds ratios (ORs) along with the 95% confidence intervals (CIs) estimated by multivariable logistic regression models

*p < 0.05, **p < 0.01, ***p < .0001

°Assessed by the Pregnancy Physical Activity Questionnaire (PPAQ) before the end of the 12^th^ week of gestation

°°Assessed by the Patient Health Questionnaire for Depression and Anxiety (PHQ)-4 before the end of the 12^th^ week of gestation

### Associations between pre-pregnancy overweight or obesity and PPD

In multivariable logistic regression analyses, the odds of experiencing PPD significantly increased with increasing BMI category. Compared to the reference weight category (normal weight), being in the overweight and obesity weight category was associated with increasing odds of PPD (Table 3; overweight: OR = 1.72, 95% CI = 1.15–2.57, *p* < 0.01; obesity: OR = 1.91, 95% CI = 1.16–3.14, *p* = 0.01). This association remained significant after adjustment for further sociodemographic or lifestyle factors, and GDM (model 3:overweight, BMI 25.0–29.9 kg/m^2^: OR = 1.78, 95% CI = 1.18–2.70, *p* < 0.01; obesity, BMI 30.0–40.0 kg/m^2^: OR = 1.80, 95% CI = 1.07–3.05, *p* = 0.03). Moreover, this association remained significant after full adjustment for antenatal history of anxiety or depressive symptoms (full model or model 4: overweight: OR = 1.72, 95% CI = 1.13–2.62, *p* = 0.01; obesity: OR = 1.76, 95% CI = 1.04–2.99, *p* = 0.04).

**Table 3:** Associations between pre-pregnancy BMI categories and PPD at 6–8 weeks postpartum (*n* = 1583)

**Model**	**BMI categories**	**OR**	**95% CI**
**1**	Normal weight		1.00 (Ref)
	Overweight	1.72	1.15–2.57**
	Obesity	1.91	1.16–3.14*
**2**	Normal weight		1.00 (Ref)
	Overweight	1.73	1.15–2.59**
	Obesity	1.83	1.10–3.03)*
**3**	Normal weight		1.00 (Ref)
	Overweight	1.78	1.18–2.70**
	Obesity	1.80	1.07–3.05*
**4**	Normal weight		1.00 (Ref)
	Overweight	1.72	1.13–2.62*
	Obesity	1.76	1.04–2.99*

Depicted are odds ratios (ORs) along with the 95% confidence intervals (CIs) estimated by multivariable logistic regression models

*Abbreviations*: *BMI* body mass index, *GWG* gestational weight gain (as defined by the IOM), *Ref* reference category

Model 1: adjusted for age and group allocation

Model 2: model 1 + marital status, educational level, and parity

Model 3: model 2 + smoking status, alcohol intake, low level of physical activity assessed by the Pregnancy Physical Activity Questionnaire (PPAQ), and gestational diabetes mellitus

Model 4: model 3 + antenatal history of anxiety/depressive symptoms during early pregnancy assessed by the Patient Health Questionnaire for Depression and Anxiety (PHQ)-4

*p < 0.05, **p < 0.01, ***p < .0001

### Association between GWG and PPD

Table 4 shows associations between GWG or excessive GWG and the odds of experiencing PPD in relation to concurrent risk factors. A 1-kg increase in total GWG increased the odds of experiencing PPD with a borderline statistical significance (Table 4, model 1: OR = 1.19, 95% CI = 1.00–1.43, *p* = 0.05). However, GWG (per 1-kg increase) was not significantly associated with PPD after adjustments for potential confounders (Table 4, model 4: OR = 1.16, 95% CI 0.94–1.44, *p* > 0.05). Further analyses disclosed that excessive GWG was significantly and positively associated with the odds of experiencing PPD when adjusted for age and group allocation (OR = 1.39, 95% CI = 1.10–1.76, *p* = 0.006). The association remained significant after adjustment for sociodemographic factors, parity, lifestyle factors, gestational diabetes mellitus, and history of anxiety or depressive symptoms during pregnancy (OR = 1.31, 95% CI = 1.06–1.61, *p* = 0.01) with history of anxiety or depressive symptoms being the most prominent determinant (OR = 3.36, 95% CI 2.46–4.58, *p* < .0001). However, excessive GWG was not independently associated with the odds of PPD when accounting for the interaction between GWG and BMI. Thus, excessive GWG was not significantly associated with PPD in the final model (Table 4, model 4: OR = 3.48, 95% CI 0.35–34.94, *p* > 0.05). The full model had the smallest QIC statistics (data not shown).

**Table 4:** Associations between (excessive) GWG and PPD at 6–8 weeks postpartum (*n* = 1583)

**Covariate**	**Model 1**	**Model 2**	**Model 3**	**Model 4**
**GWG (excessive vs. non-excessive)**	3.91 (0.41–36.90)	4.31 (0.43–42.70)	3.99 (0.42–37.90)	3.48 (0.35–34.94)
Pre-pregnancy BMI	1.07 (1.01–1.12)*	1.07 (1.01–1.13)*	1.06 (1.01–1.12)*	1.06 (1.00–1.12)
Excessive GWG * pre-pregnancy BMI	0.96 (0.88–1.05)	0.96 (0.88–1.05)	0.96 (0.88–1.05)	0.96 (0.88–1.05)
**GWG (per 1-unit increase)**	1.19 (1.00–1.43)	1.19 (0.98–1.45)	1.19 (0.98–1.44)	1.16 (0.94–1.44)
Pre-pregnancy BMI	1.12 (1.02–1.22)*	1.13 (1.03–1.24)*	1.12 (1.02–1.23)*	1.11 (1.00–1.23)*
GWG * pre-pregnancy BMI	0.99 (0.99–1.00)	0.99 (0.99–1.00)	1.00 (0.99–1.00)	1.00 (0.99–1.00)

Depicted are odds ratios (ORs) along with the 95% confidence intervals (CIs) estimated by multivariable logistic regression models

*Abbreviations*: *BMI* body mass index, *GWG* gestational weight gain, *Excessive GWG* as defined by the IOM

Model 1: adjusted for pre-pregnancy BMI, interaction term of (excessive) GWG X pre-pregnancy BMI, age, and group allocation

Model 2: model 1 + marital status, educational level, and parity

Model 3: model 2 + smoking status, alcohol intake, low level of physical activity assessed by the Pregnancy Physical Activity Questionnaire (PPAQ), and gestational diabetes mellitus

Model 4: model 3 + antenatal history of anxiety/depressive symptoms during early pregnancy assessed by the Patient Health Questionnaire for Depression and Anxiety (PHQ)-4

*p < 0.05, **p < 0.01, ***p < .0001

There was no significant evidence of a non-linear association between GWG (continuous) and PPD as calculated in the logistic regression by using restricted cubic splines (ß, standard error, *p* values: knot 1, − 0.05, 0.04, 0.17; knot 2, 0.02, 0.03, 0.42; knot 3, − 0.03, 0.05, 0.60). When GWG was assessed in three subgroups (inadequate or excessive vs. adequate (reference group)), there was a dose-response increment for the odds of having PPD, albeit non-significant (Additional file 5).

### Sensitivity analyses

**Fig. 2:**
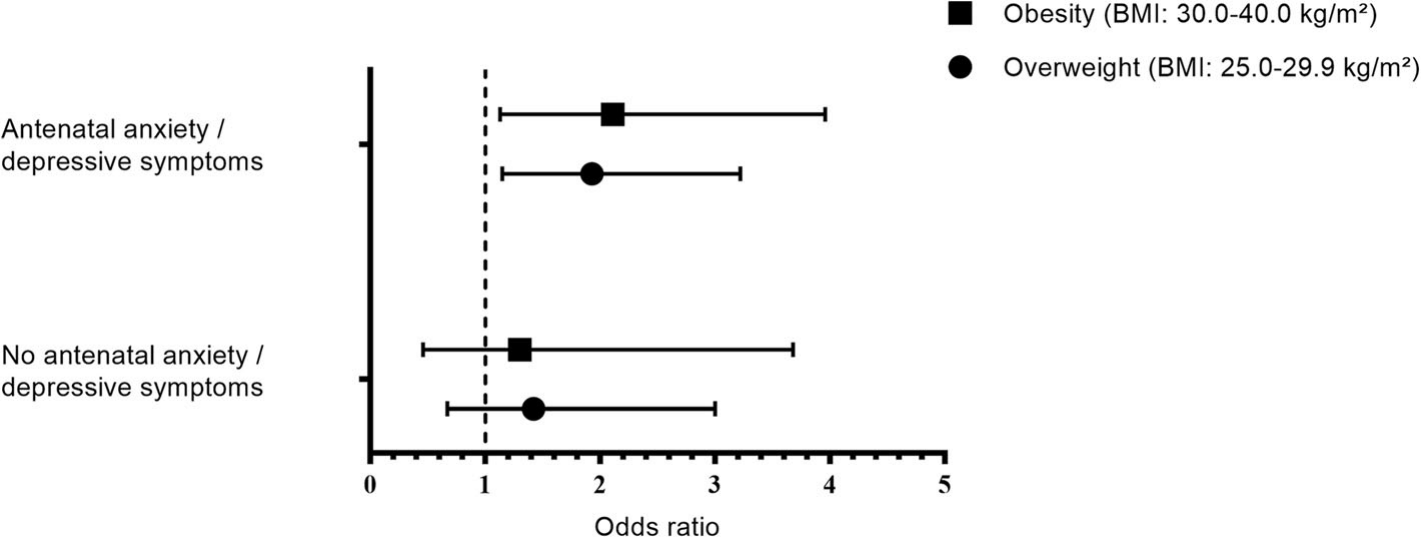
Association between pre-pregnancy overweight or obesity and PPD stratified by history antenatal anxiety or depressive symptoms. Depicted are odds ratios assessed in the fully adjusted model using logistic regression analyses controlled for the following confounders: age, group allocation, marital status, educational level, parity, smoking, alcohol intake, physical activity, and gestational diabetes mellitus as covariates. Normal weight is considered as reference category, and the corresponding odds are illustrated as dotted vertical line

We further investigated the modifying role of antenatal history of anxiety or depressive symptoms on the association between pre-pregnancy obesity and PPD, as shown in Fig. 2. The fully adjusted logistic regression model demonstrated a significant statistical interaction between antenatal history of anxiety or depressive symptoms and pre-pregnancy obesity on PPD (*p* value for interaction term = 0.03). No significant interaction of antenatal history of anxiety or depressive symptoms and GWG on PPD was observed (*p* = 0.22).

Figure 2 shows results of the logistic regression analyses on the association between pre-pregnancy BMI category and PPD stratified by antenatal history of anxiety or depressive symptoms. Pre-pregnancy overweight and obesity significantly increased the odds of experiencing PPD, but only in the subgroup of women with history of anxiety or depressive symptoms. In this subpopulation, the odds for developing PPD amounted up to 1.93 in women with overweight and 2.11 in women with obesity (fully adjusted model, overweight: OR = 1.93, 95% CI = 1.15–3.22, *p* = 0.01; obesity: OR = 2.11, 95% CI = 1.13–3.96, *p* = 0.02). Thus, both women with overweight and obesity also having a history of anxiety or depressive symptoms during pregnancy had an approximately 2-fold increased risk of experiencing PPD compared with women with normal pre-pregnancy weight and antenatal history of distress.

## Discussion

In the current analysis, including 1583 women of the GeliS trial, we evaluated the association between both pre-pregnancy BMI and GWG and the development of PPD. Additionally, we aimed to investigate a potential effect modification by an antenatal history of anxiety or depressive symptoms.

Firstly, our findings showed a significant association between pre-pregnancy BMI and the risk of experiencing PPD. This association was more pronounced when using BMI categories in comparison to a continuous BMI scale (1.76 vs. 1.23), pointing to a slight overestimation of the clinical relevance of pre-pregnancy BMI when considering only BMI categories. Our results are consistent with other research showing that a high pre-pregnancy BMI [13, 14, 21, 39], pre-pregnancy overweight [15], and obesity [14, 16] are significantly associated with having PPD. However, results are in contrast to some investigations which found no association between BMI and PPD [18, 21] or a U-shaped association with PPD [40]. To the best of our knowledge, the current study was the first to show robust effect modification by having a history of anxiety or depressive symptoms on the association between pre-pregnancy BMI and PPD. Sensitivity analyses disclosed that pre-pregnancy overweight and obesity may be potential determinants of PPD, but only in women with history of antenatal anxiety or depressive symptoms. Our results extended findings of Silverman et al. who previously reported an effect modification of having a depression history on the association between pre-pregnancy BMI and PPD among women with low BMI but not with overweight [41]. Drawing evidence from above, our results suggest a specific association between pre-pregnancy BMI and PPD in women with antenatal history of anxiety or depressive symptoms. Given the heterogeneous findings on the contribution of pre-pregnancy BMI, evidence remains inconclusive.

Secondly, our data do not provide significant evidence for an association between GWG or excessive GWG and an elevated risk for PPD in an adult population. Our findings are in line with a previous study that failed to show any association between GWG and PPD [16]. In contrast, recent findings showed a significant association between excessive GWG and PPD in adolescents who enter pregnancy with overweight or obesity [22]. Despite a high prevalence of overweight and obesity in women with excessive GWG, our analysis could not provide evidence of effect modification by pre-pregnancy overweight or obesity on the association between excessive GWG and PPD. Nevertheless, pre-pregnancy BMI seems to have a fundamental role on the interplay between excessive GWG and the risk for PPD, as the contribution of excessive GWG alone was no longer significant after adjusting for a BMI-excessive GWG interaction. Furthermore, having a history of antenatal depression or anxiety did not modify the association between GWG or excessive GWG and the risk for PPD.

Albeit women who entered pregnancy with overweight had a higher likelihood of major depression across pregnancy (up to 36^th^ week) regardless of their GWG [42], major depression during pregnancy is still thought to be more prevalent among women with GWG below the 1990 IOM recommended range [43]. Women with a BMI lower than 19.8 kg/m^2^ were previously reported to be more likely to have inadequate GWG [44]. In the GeliS study, women with a BMI below 18.5 kg/m^2^ were excluded from study participation, which may partly explain the discrepancies as we only considered the three GWG categories. Irrespective of this, we were not able to detect a significant association of either inadequate or excessive GWG and PPD in comparison to an adequate GWG. While additional adjustment for gestational age did not alter our findings (data not shown), considering trimester-specific weight gain pattern might help to disentangle heterogeneous findings on the role of excessive GWG on the risk for PPD [38].

Beyond weight-related parameters, the prospective design of the GeliS study enabled the identification of several predictors of PPD. An antenatal history of anxiety or depressive symptoms had the strongest impact on the PPD occurrence. This is in accordance with a previous review which highlighted the experience of depression and anxiety during pregnancy as the strongest predictor of PPD [45]. Silverman et al. reported a 20-fold increased risk of PPD in women with a previous history of depression compared to women without [40]. It is also likely that women with a history of antenatal depression could have a recurrent depressive disorder, and our findings show that a history of antenatal depression/anxiety may additively increase the obesity-PPD risk relationship [46, 47]. Herein, we also confirmed the consensus among systematic reviews and meta-analyses that are underlining the importance of education level and marital status as protective factors against PPD [48].

The potential underlying pathophysiological mechanism linking pre-pregnancy weight or weight changes and PPD include an elevated inflammatory state and a dysregulated hypothalamic-pituitary-adrenal axis. Published research consistently supports an association between inflammatory processes and the development of PPD [49]. Furthermore, obesity is considered as an inflammatory state [50], which may contribute to widespread immune activation, potentially exacerbating diseases associated with inflammation such as depression. There is also evidence demonstrating a stress-induced activation of the hypothalamic-pituitary-adrenal axis, with higher glucocorticoid levels leading to increased adiposity in non-pregnant populations, in particularly among women [51]. Furthermore, women with a positive history of depression are more susceptible to hormonal changes with evidence on the elevated cortisol and PPD risk [52]. ‘Stress vulnerability’ models propose that associations between pre-pregnancy weight and PPD are more pronounced among high-risk populations, in our case, among women with high BMI and history of psychological distress during pregnancy. Therefore, future work should focus on these high-risk populations by providing an appropriate prevention or intervention strategy. Moreover, it would be worthwhile to assess PPD at a later stage of the postpartum period to verify the sustainability of our findings.

### Strengths and limitations

Our study was limited by the recruitment criteria excluding women with underweight. The self-reported pre-pregnancy BMI might have led women to underreport their initial weight [53]. We acknowledge that quantitative analyses might reveal the potential contribution of bias introduced by the self-reports of pre-pregnancy weight [38, 54]. Weight during the course of pregnancy was measured in several study centres which might have introduced some inaccuracies. Through our approach of defining pregnancy weight gain with two measures (at inclusion and at birth), we did not consider trimester-specific pattern of GWG and the definite timing of exceeding IOM criteria during the course of pregnancy [38]. We acknowledge that assessing the contribution of longitudinal weight gain may provide further insights into the interplay between GWG and occurrence of PPD and might be valuable to derive concrete implications for primary care. Although the EPDS is a validated questionnaire, we are aware that estimating the rate of women with a history of depressive symptoms using the EPDS might partly underestimate the actual incidence of PPD. Despite statistical significance, our modest OR values (below 2.0) may be of moderate clinical significance and thus should be interpreted with caution.

The strength of our study is based on the trial design. Data were collected within the routine antenatal care system and thus under real-life conditions. We longitudinally collected data over the course of pregnancy and were thus able to consider the contribution of various determinants to the development of PPD beyond crude weight data. We were also able to reach a sample of participants in both urban and rural regions. The relatively large sample size provides a comprehensive and valuable assessment of early predictors of PPD. Data are robust to adjustment for an appropriate set of covariates. By employing the EPDS, we used a validated, easily applicable, and widely used screening tool for PPD symptoms.

## Conclusion

Herein, we could not provide evidence that either GWG or excessive GWG determines the risk for PPD; however, we found a significant robust association between pre-pregnancy BMI and the odds of experiencing PPD symptoms. The association was independent from various concurrent risk factors. Moreover, the influence of pre-pregnancy overweight or obesity on PPD was further amplified by an antenatal history of anxiety or depressive symptoms. Obesity and psychological distress during pregnancy may have an additive effect on the development of PPD. In addition to appropriate obesity management, health care providers should implement mental health screening strategies, both early in and throughout pregnancy, to identify women with increased risk requiring intervention to prevent PPD.

## Supplementary information

**Additional file 1: Supplementary Table 1:** PPD incidence in intervention and control groups.

**Additional file 2: Supplementary Table 2:** Characteristics (n (%)) of excluded and included study participants.

**Additional file 3: Supplementary Table 3:** Characteristics (n (%)) of GeliS study participants according to pre-pregnancy BMI categories (normal weight as reference category) (*n* = 1583).

**Additional file 4: Supplementary Table 4:** Characteristics (n (%)) of GeliS study participants according to excessive gestational weight gain (excessive vs. non-excessive) (n = 1583).

**Additional file 5: Supplementary Table 5:** Associations between GWG (inadequate, excessive vs. adequate) and PPD at 6–8 weeks postpartum (n = 1583).

## Data Availability

The datasets used and analysed during the current study are available from the corresponding author on reasonable request.

## Abbreviations

BMI: Body mass index
CI: Confidence interval
EPDS: Edinburgh Postnatal Depression Scale
GeliS: ‘Gesund leben in der Schwangerschaft’/‘healthy living in pregnancy’
GWG: Gestational weight gain
IOM: Institute of Medicine
MET: Metabolic equivalent of task
OR: Odds ratio
PHQ-4: Patient Health Questionnaire for Depression and Anxiety
PPAQ: Pregnancy Physical Activity Questionnaire
PPD: Postpartum depression
QIC: Quasi-likelihood under the independence model criterion
SD: Standard deviation
