# Supplementary material for: Evaluation of antenatal risk factors for postpartum depression: a secondary cohort analysis of the cluster-randomised GeliS trial
Source: BMC Med. 2020 Jul 24;18:227. doi: 10.1186/s12916-020-01679-7 (PMC7379365; doi:10.1186/s12916-020-01679-7)
Supplement: Supplementary file 1 — Additional file 1: Supplementary Table 1: PPD incidence in intervention and control groups. [file 12916_2020_1679_MOESM1_ESM.pdf]

Supplementary Table 1: PPD incidence in intervention and control groups.

	**Control**<br>n (%)	**Intervention**<br>n (%)	**Unadjusted OR (95% CI)**	***p***	**Adjusted OR (95% CI)***	**Adjusted *p******
No PPD	728/786 (92.6%)	717/797 (90.0%)	Ref		Ref	
PPD	58/786 (7.4%)	80/797 (10.0%)	1.36 (0.92-2.02)	0.13	1.31 (0.89-1.92)	0.17

Depicted is the odds ratio of having PPD along with the 95% confidence interval from logistic regression analyses fit with generalised estimating equations.
*Adjusted for maternal pre-pregnancy age, pre-pregnancy BMI, parity.
Abbreviations: BMI: body mass index OR: odds ratio; PPD: postpartum depression.
