# Supplementary material for: Evaluation of antenatal risk factors for postpartum depression: a secondary cohort analysis of the cluster-randomised GeliS trial
Source: BMC Med. 2020 Jul 24;18:227. doi: 10.1186/s12916-020-01679-7 (PMC7379365; doi:10.1186/s12916-020-01679-7)
Supplement: Supplementary file 2 — Additional file 2: Supplementary Table 2: Characteristics (n (%)) of excluded and included study participants. [file 12916_2020_1679_MOESM2_ESM.pdf]

Supplementary Table 2: Characteristics (n (%)) of excluded and included study participants

	Study participants (n = 1583)		Excluded participants (n = 703)		*p**
**Maternal characteristics**					
Pre-pregnancy BMI, mean ± SD	24.3 ± 4.4		24.5 ± 4.6		0.39
Pre-pregnancy BMI categories					
BMI: 18.5–24.9 kg/m²	1047	(66.1)	446	(63.4)	0.39
BMI: 25.0–29.9 kg/m²	352	(22.2)	168	(23.9)	
BMI: 30.0–40.0 kg/m²	184	(11.6)	90	(12.8)	
Excessive GWG	722	(45.6)	170	(24.2)	**0.01**
GWG, mean ± SD	14.2 ± 5.3		12.9 ± 5.4		**<.0001**
Parity					
0	930	(58.7)	369	(52.5)	**<0.01**
1	536	(33.9)	223	(31.7)	
≥ 2	117	(7.4)	83	(11.8)	
**Demographic factors**					
Age, mean ± SD	30.4 ± 4.4		30.0 ± 4.8		0.12
Educational level					
High School or others	930	(58.7)	414	(61.4)	0.24
University	653	(41.3)	260	(38.6)	
Married	1057	(66.8)	396	(56.3)	**<0.01**
Living alone	47	(3.0)	47	(7.0)	0.31
**Lifestyle and metabolic factors**					
Alcohol consumption	481	(30.4)	138	(19.6)	0.58
Smoking	80	(5.1)	48	(9.0)	**<0.01**
Low level of physical activity°	802	(51.7)	423	(60.1)	**<.0001**
Gestational diabetes mellitus	155	(9.8)	64	(9.1)	**0.01**
**First trimester psychological factors**					
Antenatal history of anxiety / depressive symptoms°°	660	(41.7)	226	(32.1)	**<.0001**

**p*-value for differences between included *vs.* excluded participants using the Kruskal-Wallis test for continuous variables and the $\chi 2$ test for categorical variables.
° Assessed by the Pregnancy Physical Activity Questionnaire (PPAQ) before the end of the 12th week of gestation.
°°Assessed by the Patient Health Questionnaire for Depression and Anxiety (PHQ)-4 before the end of the 12th week of gestation.
Abbreviations: BMI: body mass index; GWG: Gestational weight gain; Excessive GWG as defined by the IOM.
