# Supplementary material for: Evaluation of antenatal risk factors for postpartum depression: a secondary cohort analysis of the cluster-randomised GeliS trial
Source: BMC Med. 2020 Jul 24;18:227. doi: 10.1186/s12916-020-01679-7 (PMC7379365; doi:10.1186/s12916-020-01679-7)
Supplement: Supplementary file 3 — Additional file 3: Supplementary Table 3: Characteristics (n (%)) of GeliS study participants according to pre-pregnancy BMI categories (normal weight as reference category) (n = 1583). [file 12916_2020_1679_MOESM3_ESM.pdf]

Supplementary Table 3: Characteristics (n (%)) of GeliS study participants according to pre-pregnancy BMI categories (normal weight as reference category) (n = 1583).

	**Normal weight** n = 1047 (66.1%)		**Overweight** n = 352 (22.2%)		**Obesity** n = 184 (11.6%)		***p****
**Maternal characteristics**							
Pre-pregnancy BMI, mean ± SD	21.8 ± 1.8		26.9 ± 1.4		33.7 ± 3.0		**<.0001**
GWG (kg), mean ± SD	14.7 ± 4.5		14.0 ± 5.7		11.0 ± 6.7		**<.0001**
Excessive GWG	373	(35.6)	234	(66.5)	115	(62.5)	**<.0001**
Parity							
0	622	(59.4)	194	(55.1)	114	(62.0)	
1	348	(33.2)	131	(31.0)	57	(31.1)	0.56
≥ 2	77	(7.4)	27	(7.7)	13	(7.1)	
**Demographic factors**							
Age, mean ± SD	30.3 ± 4.4		30.4 ± 4.2		30.6 ± 4.8		**<.0001**
Educational level							
High School or others	571	(54.5)	230	(65.3)	129	(70.1)	**<.0001**
University	476	(45.5)	122	(34.7)	55	(29.9)	
Married	686	(65.5)	247	(70.2)	124	(67.4)	0.27
Living alone	37	(3.6)	8	(2.3)	2	(1.1)	0.13
**Lifestyle and metabolic factors**							
Alcohol consumption	315	(30.1)	109	(31.0)	57	(31.0)	0.94
Smoking	51	(4.9)	17	(4.8)	12	(6.5)	0.63
Low level of physical activity°	524	(50.1)	182	(51.7)	96	(52.2)	0.79
Gestational diabetes mellitus	70	7.0	34	(9.9)	51	(28.3)	**<.0001**
**Psychological factors**							
Antental history of anxiety / depressive symptoms°°	424	(66.1)	154	(43.8)	82	(44.6)	0.40

**p*-value for differences between pre-pregnancy obesity categories using the Kruskal-Wallis test for continuous variables and the χ2 test for categorical variables.
° Assessed by the Pregnancy Physical Activity Questionnaire (PPAQ) before the end of the 12th week of gestation.
°°Assessed by the Patient Health Questionnaire for Depression and Anxiety (PHQ)-4 before the end of the 12th week of gestation.
Abbreviations: BMI: body mass index; GWG: Gestational weight gain (as defined by the IOM).
