# Supplementary material for: Evaluation of antenatal risk factors for postpartum depression: a secondary cohort analysis of the cluster-randomised GeliS trial
Source: BMC Med. 2020 Jul 24;18:227. doi: 10.1186/s12916-020-01679-7 (PMC7379365; doi:10.1186/s12916-020-01679-7)
Supplement: Supplementary file 4 — Additional file 4: Supplementary Table 4: Characteristics (n (%)) of GeliS study participants according to excessive gestational weight gain (excessive vs. non-excessive) (n = 1583). [file 12916_2020_1679_MOESM4_ESM.pdf]

Supplementary Table 4: Characteristics (n (%)) of GeliS study participants according to excessive gestational weight gain (GWG) (excessive vs. non-excessive) (n = 1583).

	**Excessive GWG n = 722 (45.6%)**	**Non-excessive GWG n = 861 (54.4%)**	***p****
**Maternal characteristics**			
Pre-pregnancy BMI, mean ± SD	25.4 ± 4.5	23.4 ± 4.1	**<.0001**
Pre-pregnancy BMI categories			
BMI: 18.5–24.9 kg/m²	373 (51.7)	674 (78.3)	**<.0001**
BMI: 25.0–29.9 kg/m²	234 (32.4)	118 (13.7)	
BMI: 30.0–40.0 kg/m²	115 (15.9)	69 (8.0)	
Parity			
0	469 (65.0)	461 (53.5)	**<.0001**
1	207 (28.7)	329 (38.2)	
≥ 2	46 (6.4)	71 (8.3)	
**Demographic factors**			
Age, mean ± SD	29.9 ± 4.2	30.8 ± 4.6	**<.0001**
Educational level			
High School or others	465 (64.4)	466 (54.1)	**0.0003**
University	257 (35.6)	395 (45.9)	
Married	483 (66.9)	574 (66.7)	0.93
Living alone	18 (2.5)	29 (3.4)	0.31
**Lifestyle and metabolic factors**			
Alcohol consumption	213 (29.5)	268 (31.1)	0.48
Smoking	42 (5.8)	38 (4.4)	0.20
Low level of physical activity°	373 (51.7)	429 (49.8)	0.47
Gestational diabetes mellitus	54 (7.8)	101 (12.2)	**0.01**
**Psychological factors**			
Antenatal history of anxiety / depressive symptoms°°	319 (44.2)	341 (39.6)	0.07

**p*-value for differences between excessive vs. non-excessive GWG using the Kruskal-Wallis test for continuous variables and the $\chi 2$ test for categorical variables.

° Assessed by the Pregnancy Physical Activity Questionnaire (PPAQ) before the end of the 12th week of gestation.

°°Assessed by the Patient Health Questionnaire for Depression and Anxiety (PHQ)-4 before the end of the 12th week of gestation.

Abbreviations: BMI: body mass index; GWG: Gestational weight gain; Excessive GWG as defined by the IOM.
