# Supplementary material for: Evaluation of antenatal risk factors for postpartum depression: a secondary cohort analysis of the cluster-randomised GeliS trial
Source: BMC Med. 2020 Jul 24;18:227. doi: 10.1186/s12916-020-01679-7 (PMC7379365; doi:10.1186/s12916-020-01679-7)
Supplement: Supplementary file 5 — Additional file 5: Supplementary Table 5: Associations between GWG (inadequate, excessive vs. adequate) and PPD at 6–8 weeks postpartum (n = 1583). [file 12916_2020_1679_MOESM5_ESM.pdf]

Supplementary table 5: Associations between GWG (inadequate, excessive vs. adequate) and PPD at 6–8 weeks postpartum (n = 1583).

Covariate	Model 1	Model 2
GWG		
Inadequate (n = 402)	0.07 (0.01 – 0.78)	0.09 (0.01 – 1.07)
Excessive (n = 851)	1.32 (0.16 – 11.28)	1.28 (0.14 – 11.30)
Adequate (n = 632)	Ref.	Ref.
Pre-pregnancy BMI	1.03 (0.96 – 1.10)	1.03 (0.96 – 1.09)
GWG*Pre-pregnancy BMI		
Inadequate vs. adequate	1.09 (1.00 – 1.20)	1.08 (0.98 – 1.19)
Excessive vs. adequate	1.00 (0.91 – 1.09)	0.99 (0.91 – 1.09)
Age	0.97 (0.94 – 1.01)	1.01 (0.97 – 1.04)
Group effect	1.39 (0.93 – 2.10)	1.41 (0.94 – 2.12)
Married		0.69 (0.53 – 0.91)**
Lower educational level		1.32 (0.98 – 1.76)
Parity		
1		0.79 (0.57 – 1.11)
≥ 2		0.65 (0.34 – 1.23)
High alcohol intake		1.27 (0.88 – 1.84)
Low level of physical activity°		0.94 (0.71 – 1.26)
Smoking		1.49 (0.76 – 2.92)
Gestational diabetes mellitus		1.28 (0.88 – 1.87)
Antenatal history of anxiety / depressive symptoms°°		3.40 (2.45 – 4.71)***

Depicted are odds ratios (OR) along with the 95% confidence intervals (CI) estimated by multivariable logistic regression models.* $p < 0.05$, ** $p < 0.01$, *** $p < .0001$.

° Assessed by the Pregnancy Physical Activity Questionnaire (PPAQ) before the end of the 12th week of gestation.

°°Assessed by the Patient Health Questionnaire for Depression and Anxiety (PHQ)-4 before the end of the 12th week of gestation.

Abbreviations: BMI: body mass index; GWG: gestational weight gain; Excessive GWG as defined by the IOM.
